# Role of Matrix Metalloproteinases in Photoaging and Photocarcinogenesis

**DOI:** 10.3390/ijms17060868

**Published:** 2016-06-02

**Authors:** Pavida Pittayapruek, Jitlada Meephansan, Ornicha Prapapan, Mayumi Komine, Mamitaro Ohtsuki

**Affiliations:** 1 Division of Dermatology, Chulabhorn International College of Medicine, Thammasat University, Pathum Thani 12000, Thailand; p_ha_r@hotmail.com (P.P.); kae_mdcu@yahoo.com (J.M.); chad_naja@hotmail.com (O.P.); 2 Department of Dermatology, Jichi Medical University, Tochigi 329-0498, Japan; mamitaro@jichi.ac.jp

**Keywords:** matrix metalloproteinase (MMP), photoaging, photocarcinogenesis, basal cell carcinoma, squamous cell carcinoma, malignant melanoma

## Abstract

Matrix metalloproteinases (MMPs) are zinc-containing endopeptidases with an extensive range of substrate specificities. Collectively, these enzymes are able to degrade various components of extracellular matrix (ECM) proteins. Based on their structure and substrate specificity, they can be categorized into five main subgroups, namely (1) collagenases (MMP-1, MMP-8 and MMP-13); (2) gelatinases (MMP-2 and MMP-9); (3) stromelysins (MMP-3, MMP-10 and MMP-11); (4) matrilysins (MMP-7 and MMP-26); and (5) membrane-type (MT) MMPs (MMP-14, MMP-15, and MMP-16). The alterations made to the ECM by MMPs might contribute in skin wrinkling, a characteristic of premature skin aging. In photocarcinogenesis, degradation of ECM is the initial step towards tumor cell invasion, to invade both the basement membrane and the surrounding stroma that mainly comprises fibrillar collagens. Additionally, MMPs are involved in angiogenesis, which promotes cancer cell growth and migration. In this review, we focus on the present knowledge about premature skin aging and skin cancers such as basal cell carcinoma (BCC), squamous cell carcinoma (SCC), and melanoma, with our main focus on members of the MMP family and their functions.

## 1. Introduction

Skin is the primary means through which an organism interacts with its environment. Accordingly, it is regularly exposed to a direct oxidative environment, including ultraviolet (UV) radiation. Acute exposure to UV radiation causes sunburn, connective tissue deterioration, DNA injury, and immune suppression. Chronic or long-term exposure to UV radiation disrupts the normal skin structure leading to a host of skin issues including premature skin aging (photoaging) and skin cancer (photocarcinogenesis) [1,2].

UV radiation increases the expression of matrix metalloproteinases (MMPs) in human skin. MMPs are responsible for degrading the extracellular matrix (ECM) proteins such as collagen, fibronectin, elastin, and proteoglycans, contributing to photoaging [3,4]. MMPs play a crucial role in photocarcinogenesis by regulating/affecting various processes related to tumor progression including tumor institution, growth, angiogenesis, and metastasis [5].

This review focuses on human MMPs in relation to photoaging and photocarcinogenesis. The first half of the paper briefly summarizes the role of MMPs in relation to photoaging and the second half focuses on the involvement of MMPs in the pathophysiology of skin cancers such as melanoma, basal cell carcinoma (BCC), and squamous cell carcinoma (SCC).

## 2. MMPs

MMPs are zinc-containing endopeptidases with a broad range of substrate specificities. They mediate the degradation of the different components of the ECM [3,4]. They are secreted by keratinocytes and dermal fibroblasts in response to multiple stimuli such as oxidative stress, UV radiation, and cytokines [6,7]. To date, at least 28 different types of MMPs have been identified that play important roles in various pathophysiological processes including photoaging, wound healing, skeletal growth and remodeling, arthritis, inflammation, angiogenesis, and cancer [3,8,9].

MMPs can be categorized into five main subgroups based on their substrate specificity and structural organization. These are listed below in Table 1:

1. Collagenases (MMP-1, MMP-8, and MMP-13) recognize the substrate through a hemopexin-like domain and are able to degrade fibrillar collagen [10].

2. Gelatinases (MMP-2 and MMP-9) are able to digest a number of ECM components such as collagen type I and IV.

3. Stromelysins (MMP-3, MMP-10, and MMP-11) have a domain arrangement similar to that of collagenases; however, they do not cleave fibrillar collagen type I.

4. Matrilysins (MMP-7 and MMP-26) lack a hemopexin-like domain and degrade collagen type IV but not type I.

5. MT-MMPs (MMP-14, MMP-15, and MMP-16) have an additional C-terminal transmembrane domain with a short cytoplasmic tail. Both MMP-14 and MMP-16 degrade fibrillar collagen type I. 

In addition to the five aforementioned subgroups of MMPs, there are few MMPs that are not grouped into any of these categories, such as metalloelastase (MMP-12), RASI-1 (MMP-19), enamelysin (MMP-20), and epilysin (MMP-28) [9].

## 3. Photoaging

Aging changes in the skin can be categorized in two groups: (1) intrinsic, or chronologic aging, an inherent degenerative process due to declining physiological functions and capacities; and (2) extrinsic, or photoaging, a distinctive deteriorating process caused by environmental factors. UV radiation is the major environmental factor that causes photoaging [11,12]. The action spectrum for UV-induced skin damage is divided into UV-A (320–400 nm) and UV-B (290–320 nm). UV-A rays account for up to 95% of the UV radiation reaching the Earth’s surface and is only slightly affected by ozone levels. The amount of UV-B reaching the earth’s surface is lesser than that of UV-A; however, its intensity is high enough to cause photoaging and skin cancer [13,14]. Nonetheless, both UV-A and UV-B irradiation can induce oxidative stress in human skin, leading to temporal and persistent genetic impairment, up-regulation of activator protein (AP)-1 activity, and increased MMP expression (Table 2) [15,16].

Photoaging involves prominent cutaneous transformation that is clinically characterized by fine and coarse wrinkles, blotchy dyspigmentation, telangiectasia, sallowness, increased fragility, and rough skin texture [3]. Additionally, histological and ultrastructural studies have revealed epidermal hyperplasia, damaged and disorganized collagen fibrils, and substantial accumulation of abnormal elastic material in dermal connective tissue [16,17].

Photoaging is caused by an imbalance in equilibrium between the accumulation and degradation of ECM components that provide structural and functional support to the skin tissue. Cumulative exposure to the sun results in continuous degradation of ECM proteins such as collagen and elastin, and a decreased rate of renewal/synthesis of collagen. Collagen is the primary insoluble fibrous protein in the ECM and in connective tissue. Type I collagen is the most abundant subtype of collagen found within connective tissue of the skin, followed by small amounts of type III collagen. Fibroblasts, located within the dermis, mainly synthesize collagen, which imparts strength and elasticity to the skin [12,14,17].

**Figure 1:**
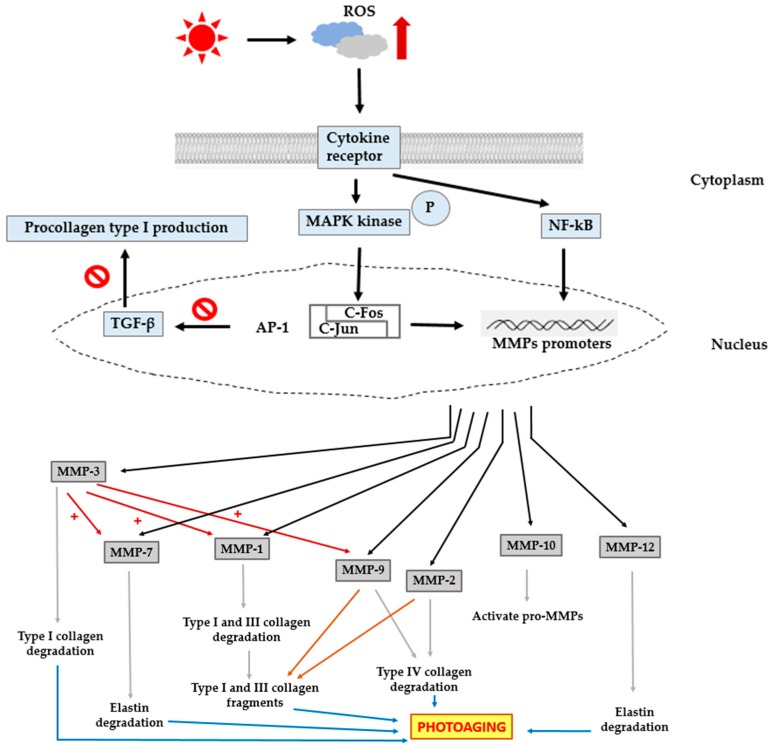
Schematic diagram showing the role of MMPs in photoaging. UV-induced excess intracellular reactive oxygen species (ROS) activates mitogen-activated protein kinases (MAPKs) and nuclear factor-kappa B (NF-κB), culminating in the transcriptional regulation of MMPs, and results in the degradation of collagen and elastin, subsequently leading to photoaging. In addition to collagen and elastin degradation, AP-1 inhibits transforming growth factor beta (TGF-β) signaling, causing a reduction in procollagen synthesis.

Degradation of collagen is normally regulated by MMPs and by the activity of their natural inhibitors, tissue inhibitor of metalloproteinases (TIMPs). Increased MMP activity is an important factor influencing the development of age-related changes in skin [18] (Figure 1 and Table 1). 

In the skin, epidermal keratinocytes and dermal fibroblasts mainly secrete MMP-1(interstitial collagenase or collagenase 1), a collagenase that degrades fibrillar collagens type I and III into specific fragments at a single site within the central triple helix. Other MMPs such as gelatinases, further hydrolyze these fragments, ultimately impairing the function of the collagen-rich dermis [1,4,8,19].

UV irradiation induces increased synthesis and expression of MMP-1 by dermal fibroblasts, which is stimulated by the generation of excess reactive oxygen species (ROS), and plays a critical role in photoaging. UV irradiation induces excess intracellular ROS such as singlet oxygen (^1^O_2_), superoxide anion (O_2_^−^), hydrogen peroxide (H_2_O_2_), and hydroxyl radicals (OH^.^) [17]. ROS, a secondary messenger, activates the mitogen-activated protein kinase (MAPK) family. MAPKs are a family of proline-directed Ser/Thr kinases comprising extracellular signal-regulated kinases (ERKs), p38, and c-Jun NH2-terminal kinase (JNK). ERK is important to stimulate the expression of c-Fos, whereas p38 and JNK activation are crucial for the expression of c-Jun. c-Jun in combination with c-Fos forms the transcription factor AP-1, which plays an essential role in the transcriptional regulation of MMP-1, MMP-3, and MMP-9 resulting in the degradation of collagen [4,17,20]. Additionally, AP-1 inhibits transforming growth factor-β (TGF-β) signaling, a major regulator for the production of procollagen type I in human skin. Impairment of the TGF-β pathway leads to decreased synthesis of procollagen [21,22,23]. Besides AP-1, nuclear factor-kappa B (NF-κB) is another important transcription factor that is activated in response to UV irradiation. NF-κB is a universal transcription factor that regulates the gene expression of growth factors, chemokines, cytokines, and cell adhesion molecules, in healthy as well as numerous diseased states. Generation of ROS induces NF-κB-mediated transcriptional activation and regulation of MMP gene expression. Thus, this factor is important to mediate the responses of UV irradiation. NF-κB activity is reported to be responsible for the up-regulation of MMPs such as MMP-1 and MMP-3 in dermal fibroblasts [2,20,24]. Thus, both AP-1 and NF-κB are involved in the process of photoaging.

UV-induced AP-1 activation enhances the expression of MMP-1, MMP-3, and MMP-9. MMP-3, known as stromelysin-1, differs from collagenases because of its inability to digest collagen type I. However, it does degrade a large number of ECM proteins, such as type IV, V, IX, and X collagens, gelatin, fibrillin-1, fibronectin, laminin, and proteoglycans. The primary function of MMP-3 is the activation of pro-MMPs such as collagenases, gelatinase B, and matrilysins during ECM turnover. In particular, the production of fully active MMP-1 MMP-3 is essential to partially activate pro-MMP-1 [9,25,26]. MMP-10, known as stromelysin-2, cleaves various ECM proteins and is involved in the activation of pro-MMPs. However, the catalytic function of collagen type IV and type V is quite weak compared to the MMP-3 activity [9,27].

MMP-9, known as gelatinase B or 92-kDa type IV collagenase, is a member of the gelatinase subgroup of MMPs, whose expression is largely dependent on the activation of AP-1. MMP-9 is produced by human keratinocytes and can digest collagen type IV, an important component of the basement membrane in skin. The epidermal basement membrane is responsible for the epidermal-dermal adhesion, which is crucial for epidermal integrity. It is also important in controlling epidermal differentiation [8,9,28,29]. Like MMP-9, MMP-2 (known as gelatinase A or 72-kDa type IV collagenase) is able to cleave collagen type IV [30]. Additionally, both these gelatinases can degrade other substrates such as collagen type V, VII, and X, fibronectin, and elastin. They are essential in degrading fibrillar collagen fragments after their initial degradation by collagenases [12,25,31].

Collagenases refer to a class of MMPs with the ability to degrade native collagen without unwinding the triple helical assembly of the substrate. Interstitial collagenase (MMP-1), neutrophil collagenase (MMP-8), and collagenase 3 (MMP-13) belong to this group [9]. They have similar configuration and enzymatic functions, despite small differences in substrate specificity. As mentioned above, MMP-1 plays an important role in the photoaging process. Recent studies suggest a limited role for MMP-8 in UV-mediated collagen damage in the skin. Although this enzyme was found to be induced by UV light, it is up-regulation was minimal [32]. MMP-13 shows higher cleavage specificity for collagen type II, a major collagen present in the cartilage, compared to collagen type I and III. MMP-13 is five times less potent than MMP-1 in cleaving collagen types I and III; however, it is 5–10 times more potent in cleaving collagen type II [9]. Hence, during photoaging, MMP-8 and MMP-13 probably contribute very little to the overall structural damage to collagen.

In addition to the degradation of collagens in skin, changes in the level of elastin have also been well documented in the process leading to photoaging. Elastin is a major component that contributes to the function of recoil and resilience, although it constitutes only 2%–4% of the total protein content of the skin. Reduced levels of elastin are associated with various diseases such as atherosclerosis and arthritis. Degradation of elastin results in an aged appearance of the skin [33,34,35]. MMP-12, known as macrophage metalloelastase, is the most effective MMP against elastin. Macrophages and fibroblasts secrete MMP-12 in response to acute UV radiation. MMP-12 plays a crucial role in the development of solar elastosis as indicated by the association between the expression of MMP-12 and the amount of elastotic material in the upper dermis of photodamaged skin [30,33,35]. The process of solar elastosis refers to the collection of dystrophic elastotic material in the dermis [15,33]. In addition to elastin, MMP-12 can cleave many other substrates belonging to the ECM, such as collagen type IV fragments, fibronectin, fibrillin- 1, laminin, entactin, vitronectin, heparin, and chondroitin sulfates. MMP-12 is also responsible for the activation of other pro-MMPs, such as pro-MMP-1, MMP-2, MMP-3, and MMP-9 [9,34]. In addition to MMP-12, MMP-7 (called matrilysin) can efficiently degrade elastin. Upon UV irradiation, MMP-7 can cleave not only elastin but also many other substrates of the ECM, such as collagen type IV, entactin, fibronectin, laminin, and cartilage proteoglycan aggregates [9,30].

Neutral endopeptidase (NEP) or neprilysin, a 94-kDa membrane-bound type of metalloprotease, is identical to fibroblasts-derived elastase. It exhibits similarities in terms of its membrane-bound metalloproteinase nature and inhibitory profiles [36,37]. The enhanced NEP activity in dermal fibroblasts plays an important role in the UV-B-induced cascade of biological processes that lead to skin wrinkling and/or sagging. This occurs because of the deterioration of the three-dimensional structure of elastic fibers and the subsequent loss of skin elasticity [36,38]. The expression of NEP is associated with keratinocyte-derived cytokines including interleukin-1 alpha (IL-1α) and granulocyte macrophage colony stimulatory factor (GM-CSF). Repetitive exposure to UV-B radiation activates keratinocytes to secrete IL-1α**,** which then stimulates the secretion of GM-CSF in an autocrine manner. Additionally, UVB can directly stimulate keratinocytes to produce GM-CSF. Both, IL-1α and GM-CSF enter the dermis and stimulate fibroblasts to up-regulate NEP. NEP then destroys the three-dimensional architecture of the elastic fibers thereby impairing skin elasticity, resulting in wrinkle formation in the skin [36,38].

MMPs play a significant role in wrinkle formation, a characteristic of photoaging. Evolution of novel MMP inhibitors is promising as targets to combat photoaging. In recent years, there has been considerable interest in the use of botanical supplements for the prevention of solar UV radiation-induced skin photodamage (Table 3). *Galla chinensis*, a natural traditional Chinese medicine, is known to significantly suppresses UV-B-induced ROS and MMP-1 expression in normal human dermal fibroblasts [21]. Extracts of *Neonauclea reticulata*, a member of Rubiaceae, a flavonoid-enriched flowering plant, significantly decreases the expression of MMP-1, MMP-3, and MMP-9 by suppressing ERK, p38, and JNK phosphorylation. *Ixora parviflora* and *Coffea arabica*, polyphenol-enriched members of Rubiaceae family, exhibit anti-photoaging activity by inhibiting the expression of MMP-1, MMP-3, and MMP-9, and MAPK activity [12,17,39].

Another strategy to diminish the damaging effects of UV radiation on skin is the use of antioxidants or free radical scavengers (Table 3). Polyphenols, with a higher number of OH groups act as ROS scavengers and protect against cellular damage. Natural products with high polyphenol contents such as *Emblica officinalis*, *Coffea arabica*, *Terminalia catappa*, and epigallocatechin-3-gallate (EGCG) render effective protection against photoaging [14,17,31]. In addition to phenolic compounds, coriander leaf extract, *Gynura procumbens*, and *Caesalpinia sappan* L. also exhibit strong protective effects against UV-induced oxidative stress [4,22,24].

## 4. Photocarcinogenesis

### 4.1. BCC

Overexposure of human skin to solar UV radiation is a major environmental risk factor for melanoma and nonmelanoma skin cancers [40]. Nonmelanoma skin cancers include BCC and SCC, which are responsible for approximately 80% and 20% of all nonmelanoma skin cancer cases, respectively [41,42,43].

In humans, skin neoplasms commonly arise in the epidermis or hair follicles where proliferating basaloid tumor cells are present. To date, most of the studies on BCC are based on Caucasian populations, as this type of cancer is less common in Asians and Black African races. It has been shown that the incidence of BCC in Asian individuals range from 16 to 20 per 100,000, and is increasing since the 1960s. In contrast, the incidence of BCC in Caucasians has been approximated to be higher than 200 and 400 per 100,000 in females and males, respectively [44,45].

**Figure 2:**
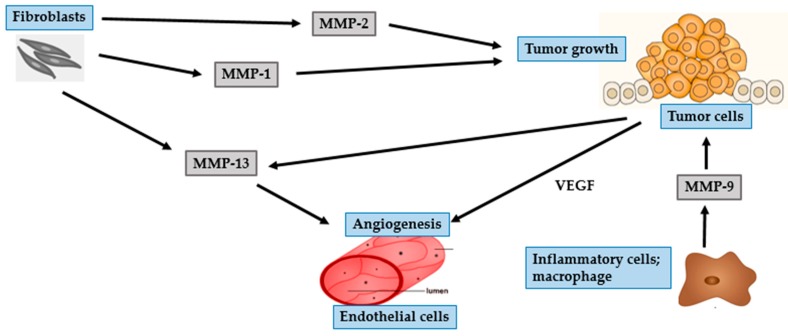
Role of MMPs in BCC and tumor growth. MMP-1 and MMP-2 secreted by fibroblasts facilitate tumor growth. MMP-13 secreted by fibroblasts and tumor cells promote tumor angiogenesis. MMP-9 secreted by inflammatory cells activates BCC cells to secrete VEGF, which in turn promotes angiogenesis.

**Figure 3:**
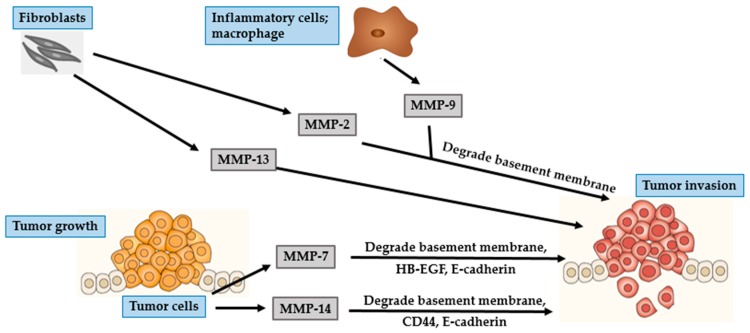
Role of MMPs in BCC tumor invasion. MMP-2 and MMP-13 secreted by fibroblasts facilitate tumor invasion. Inflammatory cells-derived MMP-9 degrades the basement membrane and promotes tumor invasion. BCC cells secrete MMP-7 and MMP-14. MMP-7 degrades the basement membrane, HB-EGF and E-cadherin, whereas, MMP-14 degrades the basement membrane, CD-44 and E-cadherin. Together, they play an important role in tumor invasion.

BCC mostly occurs in parts of the body that are frequently exposed to the sun, such as the head and neck regions. In particular, BCC often develops on the face with the nose and the lip being the most commonly affected areas [42,46]. Though BCC is characterized by slow progression and low metastatic potential, it has a propensity to be locally destructive. If untreated, BCC may invade subcutaneous fat, muscles, and even bones [42,47,48]. The invasion of tumor cells is a complex, multistage process, which starts with proteases such as MMP degrading the basement membrane and the ECM surrounding the original tumor (Figure 2 and Figure 3), altering cell-to-cell adhesion properties, reorganizing the ECM surroundings, suppressing anoikis, and rearranging the cytoskeleton to facilitate cell motility. These processes are governed by complex interactions between various biomarkers, especially MMPs, cell–cell adhesion molecules (such as β-catenin), and chemokine receptor-ligand complexes (SDF- 1/CXCR4) [42,47,49].

Two essential steps in tumor development are degradation of the basement membrane and invasion of the surroundings tissue by tumor cells [43,46,47,50]. Gelatinases are known to be associated with cancer invasion because of their ability to degrade crucial components of the basement membrane, especially collagen type IV [43,50]. MMP-2 is mostly secreted by fibroblast-like stromal cells surrounding BCC tumors, and rarely by keratinocytes and BCC tumor cells. Interaction of stromal fibroblasts and BCC tumor cells affects fibroblasts-derived MMP-2, suggesting a significant effect of this interaction in the development of cancer [5,51]. MMP-2 plays an important role in creating a suitable microenvironment for the proliferation of cancer cells and contributes to epithelial–mesenchymal transition (EMT). This transition depends upon the eliminating of adhesion molecules, such as cadherins and integrins, and a marked reorganization of the cytoskeleton, both of which facilitate the separation of malignant cells from the primary tissue. All of these processes are associated with the activity of MMP-2 [9].

MMP-9, is a gelatinase with proteolytic activity against the basement membrane components, including collagen type IV [5,41]. MMP-9 is mostly secreted by inflammatory cells such as macrophages, rather than by tumor cells [46,52]. Macrophages located within the tumor environment are called tumor-associated macrophages (TAMs). TAMs are a type of M2 macrophage that supports tumor growth. In addition to secreting proteases, TAMs can activate COX-2 in BCC cells. Overexpression of COX-2 induces secretion of angiogenic factors such as vascular endothelial growth factor (VEGF), and basic fibroblast growth factor (bFGF) [53].

Inflammatory cells surrounding BCC are typically positive for MMP-13, MMP-1, and MMP-9, indicating an essential role of inflammation in modulating tumor progression [42]. MMP-13 is involved in the degradation of ECM and its expression is associated with malignant transformation in skin carcinogenesis [42,46,47]. The expression of MMP-13 is not confined to tumor cells alone, as its expression is up-regulated in stromal cells surrounding epithelial tumors including fibroblasts, inflammatory cells, and endothelial cells. Endothelial cells-derived MMP-13 is associated with endothelial cell proliferation and vascular differentiation [42]. Of the other collagenases, MMP-1 is the primary collagenolytic enzyme in BCC. The expression of MMP-1 is significantly enhanced by fibroblasts at the invasive front of BCC, suggesting its role in the initial steps of tumor proliferation, which is mediated by cleaving the ECM proteins and active forms of growth factors that subsequently stimulate cancer cells [46,51].

UV irradiation increases tyrosine phosphorylation of β-catenin. The epidermal growth factor receptor (EGFR) activation signaling pathway facilitates nuclear translocation of phosphorylated β-catenin. In the nucleus, β-catenin regulates the Wnt/T-cell transcription factor (TCF) signaling, thereby stimulating gene transcription of MMPs, including MT1-MMP and matrilysin [42,48]. MT-MMPs are different from other soluble MMPs because of the presence of an additional C-terminal transmembrane domain and a short cytoplasmic tail. MT1-MMP or MMP-14, a classical type of MT-MMP, acts as a membrane activator of other soluble MMPs, such as MMP-2 [43,48]. Expression of MT1-MMP is involved in the degradation of ECM barrier, which then promotes tumor invasion because of its localization at the invasive front of tumor invading cells. In addition to its ability to degrade multiple components of the ECM, MT1-MMP can degrade cell adhesion molecules and signaling receptors such as CD44 and E-cadherin [48]. E-cadherin, a calcium-dependent cell adhesion protein, plays a primary role in intercellular adhesion in all the layers of the epidermis except the outermost layer [54]. CD44, a cell-surface glycoprotein, is a member of the hyaluronate receptor of cell adhesion molecules [55]. Loss of either CD44 or E-cadherin leads to impairment of epidermal cell adhesion, thereby promoting invasion of malignant tumor cells into the neighboring tissues [52].

MMP-7 or matrilysin is a widespread metalloproteinase that can degrade numerous ECM and cell surface proteins including E-cadherin and heparin-binding epidermal-like growth factor (HB-EGF) precursor. CD44 mediates the recruitment of active MMP-7 and HB-EGF precursor to form a complex on the surface of tumor cells. In this complex, MMP-7 processes HB-EGF precursor and the resultant HB-EGF activates and stimulates its receptor, ErbB4, resulting in the destruction of the basement membrane, which is an essential step towards tumor progression [52]. MMP-26, also known as matrilysin-2 or endometase, is the smallest known MMP to date. Its expression is barely detected in BCC epithelium or stromal cells and is therefore not considered significant in the development of BCC nor in the process of angiogenesis [46,56].

### 4.2. SCC

The second most common type of nonmelanoma skin cancer is SCC, accounting to approximately 20% of all skin malignancies [57,58,59]. It is characterized by malignant proliferation of epidermal keratinocytes. Causes for the development of SCC are multifactorial, including both host and environmental factors. The common host risk factors include genetic predisposition, immunosuppression, human skin type, and human papilloma virus infection. The common environmental risk factors include UV exposure, ionizing radiation, exposure to certain chemicals such as arsenic, and smoking. UV radiation is considered the predominant risk factor for SCC [57,58,59]. Unlike BCC, SCC exhibits an increased risk of metastasis, though the rate of metastasis is much lower than that of melanoma [58,60].

**Figure 4:**
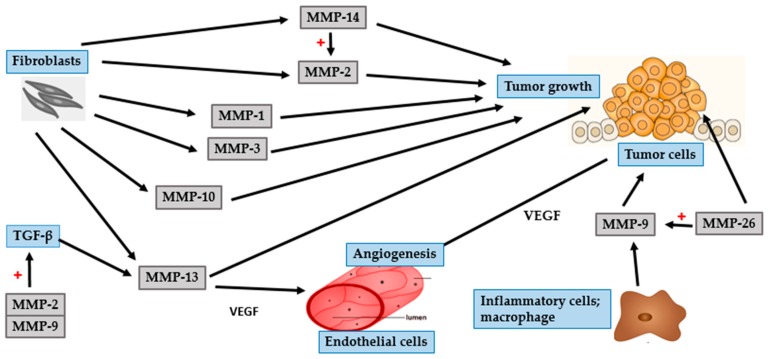
Role of MMPs in SCC and tumor growth. Fibroblasts secrete MMP-1, MMP-2 (also activated by MMP-14), MMP-3, MMP-10, MMP-13 (also enhanced by TGF-β that is activated by MMP-2 and MMP-9), and MMP-14 to promote tumor growth. In addition to tumor initiation, MMP-13 is involved in the maintenance of angiogenesis through the release of VEGF. Inflammatory cells-derived MMP-9 induces SCC cells to release VEGF to support tumor angiogenesis. MMP-26 can activate MMP-9 and promote tumor growth.

**Figure 5:**
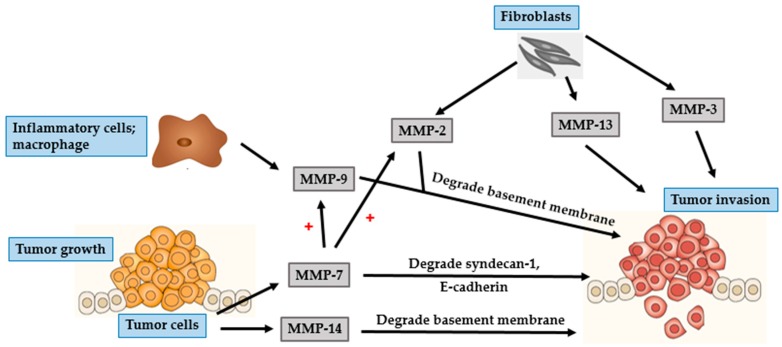
Role of MMPs in SCC tumor invasion. Fibroblasts secrete MMP-2, MMP-3, and MMP-13 to promote tumor invasion. Inflammatory cells secrete MMP-9 that degrades the basement membrane, leading to tumor invasion. SCC cells secrete MMP-7, which degrades syndecan-1, E-cadherin and induces the expression of MMP-2 and MMP-9. Additionally, SCC cells secrete MMP-14, which degrades the basement membrane. The expression of both MMP-7 and MMP-14 are important for tumor invasion.

Metastasis of cancer cells is a complex multistep process involving altered cell-to-cell adhesion, degradation of the ECM and basement membrane, detachment of tumor cells from the original site, intravasation into lymphatic or blood vessels, and establishment of new tumor at distant sites [60,61,62]. The barrier that mainly restricts development of cancer by preventing tumor invasion and metastasis is composed of the ECM and basement membrane. Thus, it is well established that the degradation of ECM and basement membrane, which requires a wide range of proteolytic enzymes, enhance the ability of tumor cells to invade and metastasize. MMPs play an important role in this process and as they have been implicated in the degradation of ECM and basement membrane [60,63] (Figure 4 and Figure 5).

MMP-2 and MMP-9, members belonging to the gelatinase subgroup, play an essential role in SCC invasion and metastasis. This ability is mainly attributed to the cleavage of collagen type IV, the major component of the basement membrane. Additionally, gelatinases function to regulate the activity of numerous growth factors and cytokines. This affects immune response and angiogenesis, leading to the proliferation and maintenance of primary and metastatic tumors [63,64,65]. The greater invasive property of SCC, compared to that of BCC, might be due to the enhanced expression and activity of gelatinases [5,43]. Gelatinolytic activity is initiated at the onset of invasive growth, mainly in the tumor stroma. Fibroblasts are potent producers of MMP-2 [64]. In contrast, neutrophils, mast cells and macrophages are the predominant source of MMP-9 [5,65]. Fibroblast-derived MMP-2 is expressed to a greater degree during the earlier stages of squamous carcinogenesis, resulting in the initiation of tumor growth. Inflammatory cell-derived MMP-9 promotes tumor invasion and angiogenesis by mediating the release of TGF-β and VEGF [64,66,67]. The expression of VEGF is also governed by hypoxia-inducible factor-1α (HIF-1α). During tumor progression, the proliferating tumor cells increase oxygen consumption, thereby worsening hypoxia. In order to adapt to the hypoxic condition, tumor cells up-regulate the expression of HIF-1α, which further aids tumor development and angiogenesis [68,69].

Similar to MMP-2, the collagenases MMP-1 and MMP-13 are expressed in stromal cells, particularly in tumor-associated fibroblasts [61,70]. Both MMP-1 and MMP-13 are able to cleave native fibrillar collagen, an important step in tumor invasion and metastasis [67,71]. Expression of MMP-1 is reported to be associated with the initial steps of tumor growth in cutaneous SCC [72]. The involvement of cytokines in the initiation and development of tumor is an interesting fact in tumor biology. Interleukin (IL)-6, a potent pleiotropic cytokine, produced by various cell types such as activated keratinocytes, B- and T lymphocytes, macrophages, and endothelial cells, has a variety of biological functions. IL-6 is a key factor driving tumor progression [72]. The expression level of IL-6 is related to the expression of MMP-1. This indicates a role for MMP-1 in the regulation of cytokine and protease network, particularly related to SCC tumor progression [59]. Thus, overexpression of MMP-1 correlates positively with tumor aggressiveness and a poor clinical outcome.

MMP-13 has great substrate specificity and is a powerful tool for tumor invasion [73]. It is mainly produced by stromal cells that lie in close vicinity to tumor cells, which supports the crucial role of stroma in tumor progression. Furthermore, MMP-13 is involved in the maintenance of angiogenesis through the release of VEGF from the tumor ECM [67,71]. The level of MMP-13 is up-regulated by a multifunctional growth factor called TGF-β, which exerts various effects on ECM deposition, tumor cell proliferation and progression [73]. Interestingly, TGF-β is activated by gelatinolytic enzymes (MMP-2 and MMP-9) [61]. Taken together, tumor cell invasion and metastasis is mediated by the interaction between various MMPs and growth factors.

Several other MMPs are reported to be involved in the pathophysiology of SCC. Among them, MT1-MMP or MMP-14 acts as the most powerful pericellular proteolysis mediator. Tumor cells are controlled by processes that help them to pass through the ECM and to migrate and invade in the form of a single cell and as a collective tumor in a greatly arranged manner [61]. MT1-MMP, a member of the MT-MMP subgroup, plays an important role in the degradation of various ECM proteins and in activating pro-MMP-2. Both stromal fibroblasts and tumor cells in SCC, particularly at the invasive front of the tumor, secrete MMP-14. This finding suggests that the expression of MMP-14 in fibroblasts and tumor cells are related to tissue remodeling and invasive tumor growth, respectively [57,64]. The aggressive behavior of SCCs is illustrated by the fact that MMP-14 is expressed at the surface of tumor cells.

Although MMP-7 is the smallest member of the MMP family, it is able to digest a wide range of ECM proteins and cleave several cell surface proteins including-cadherin and syndecan-1 [50,54]. E-cadherin/syndecan-1 complex is a potent suppressor of invasion, and loss of E-cadherin or syndecan-1 on the cell surface leads to epithelial cell transformation [54]. In addition to enhancing tumor invasion and metastasis directly, MMP-7 exerts indirect effects through the activation of MMP-2 and MMP-9 [50]. Unlike several other MMPs that are involved in varied stages of tumor invasion, MMP-26 plays an essential role in the initial stages of skin cancer. MMP-9, the most important MMP in SCC tumor growth, is stimulated by MMP-26 [65].

MMP-10 or stromelysin-2, similar to other metalloproteases, facilitates the recruitment of infiltrating cells by remodeling the ECM. Moreover, MMP-10 up-regulates several other MMPs such as MMP-1, MMP-7, MMP-9, and MMP-13 that are essential for tumor progression. The function of this protease is restricted to the initial process of tumor initiation, indicating that it might not be important in invasion or metastasis [74]. MMP-10 is highly expressed in SCC stromal cells and is up-regulated by tumor-associated cytokines including TGF-β and TNF-α. Another stromelysin, MMP-3 is induced in the tumor stroma in the early stages of tumorigenesis. It can degrade a variety of matrix and non-matrix molecules such as growth factors, HB-EGF, and E-cadherin. Fibroblasts-derived MMP-3 is a necessary mediator of tumor vascularization and tumor progression, and thus plays an important role in mechanisms that modulate tumor metastasis [70,75].

Since MMPs appear to play a crucial role in the pathogenesis of SCC, many researchers have tried to develop specific inhibitors for each MMP as targeted therapeutics for the treatment of cancer. Vitamin D3 analogue, calcipotriol, down-regulates the expression of MMP-9 and MMP-13 in normal human epidermal keratinocytes and human squamous-cell-carcinoma cell line [76]. Hence, suppressive effects of calcipotriol on the expression of MMP-9 and MMP-13 could be considered as a strategy for cancer treatment.

An natural compound,α-mangostin, a xanthone compound isolated from the pericarp of mangosteen, suppresses the activity of gelatinases, thus inhibiting the processes involved in metastasis [60].

### 4.3. Malignant Melanoma

Cutaneous melanoma, a type of cancer arising from pigment-producing cells called melanocytes, is known for its rapid progression, metastasis, and high morbidity and mortality in patients [77,78]. It accounts for 3%–-mangostin, a xanthone compound isolated from the pericarp of 5% of all cutaneous cancers, 75% of all skin cancer mortality, and has a 5-year survival rate lower than 15% for metastatic cases [77,78,79]. Development of melanoma is divided into two phases, radial growth phase (RGP) and vertical growth phase (VGP) or metastatic phase [79,80]. In the early stages of melanoma, RGP exists solely within the epidermis, with only a small number of non-dividing cells present within the superficial dermis. VGP melanomas may develop directly from RGP melanomas and are characterized by the invasion of dermal and subcutaneous tissues with the ability to disperse to regional lymph nodes and distant organs [80,81].

**Figure 6:**
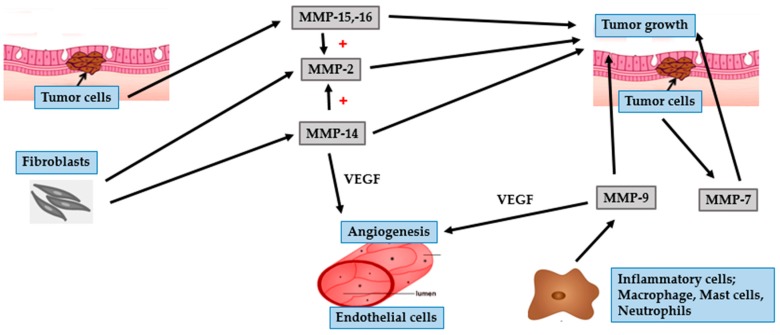
Role of MMPs in malignant melanoma and tumor growth. Melanoma cells secrete MMP-15 and MMP-16 to promote tumor growth. Fibroblasts secrete MMP-2 (also activated by MMP-14, MMP-15, and MMP-16) and MMP-14 to induce tumor growth. In addition to growth initiation, MMP-14 promotes tumor angiogenesis by enhancing the expression of VEGF. Inflammatory cells-derived MMP-9 is involved in tumor development and tumor angiogenesis. MMP-7 produced by melanoma cells can enhance tumor growth.

**Figure 7:**
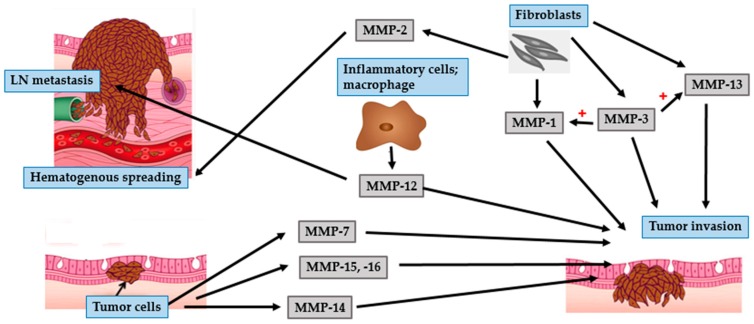
Role of MMPs in malignant melanoma tumor metastasis. Fibroblasts secrete MMP-1, MMP-3 (also activate MMP-1 and MMP-13) and MMP-13 to facilitate tumor invasion. Melanoma cells secrete MMP-7, MMP-14, MMP-15, and MMP-16 and facilitates tumor invasion. Inflammatory cells secrete MMP-12 to promote tumor invasion and are involved in lymph node (LN) metastasis. Fibroblasts-derived MMP-2 correlates with hematogenous spreading.

Like nonmelanoma skin cancers, the activity of MMPs can promote invasion of melanoma cells by altering the basement membrane and ECM proteins [77] (Figure 6 and Figure 7). The gelatinases, MMP-2 and MMP-9, primarily cleave collagen type IV and are believed to play a crucial role in the progression of melanoma cells [78]. Fibroblasts secrete pro-MMP-2 which is activated by MMP-14 [82]. Overexpression of MMP-2 is correlated with architectural impairment, atypia progression, and hematogenous metastasis [83,84]. MMP-9 is strongly expressed in inflammatory cells such as macrophages, neutrophils, and mast cells. Its expression is related to the RGP rather than the VGP of melanomas, and is probably associated with early stages of tumor invasion [83,84].

Angiogenesis is the initial process involved in the transition from a pre-neoplastic to a neoplastic stage [85]. Hypoxia is a strong stimulus for tumor angiogenesis. Tumor cells become hypoxic as they become distant from nearby vessels and respond to hypoxia-related angiogenic factors. HIF-1α, a master regulator of cellular hypoxic response, is a transcription factor essential for the transcriptional activation of VEGF, resulting in tumor angiogenesis [86,87]. In addition to angiogenesis, under hypoxic conditions, HIF-1α plays an important role in the metastatic progression of melanoma by enhancing the expression of MMP such as MMP-2, MMP-9, and MMP-14 [87,88].

An adequate supply of oxygen and nutrients for tumor development, and a route for tumor cell migration and invasion, are highly correlated with tumor angiogenesis. Many angiogenic factors are secreted by tumor cells, including VEGF, bFGF, and platelet-derived growth factor (PDGF). The most essential pro-angiogenic factor is VEGF and its level is related to tumor vascularization [89,90]. Expression of MMP-9 plays an important role in tumor angiogenesis as it enhances the availability of VEGF in malignant tumors. VEGF can up-regulate MMP-2 expression in melanoma cells [89,90]. Taken together, interaction between gelatinases and VEGF promote tumor progression by regulating tumor angiogenesis and metastasis.

MT1-MMP plays a primary role in angiogenesis by promoting the expression of VEGF, mediating endothelial cell migration and vascular formation processes [91,92]. Furthermore, MT1-MMP cleaves a wide range of ECM components, promote tumor development and invasion, and activate pro-MMP-2 on the surface of melanoma cells [84,85]. Thus, MT1-MMP is crucial for tumor invasion and for activating pro-tumorigenic MMPs. Of the other MT-MMPs, the expression of MT2-MMP (MMP-15) and MT3-MMP (MMP- 16) is increased in primary and metastatic melanoma cells [92]. Like MT1-MMP, MT3-MMP is also an efficient cell surface activator of pro-MMP2 [93].

Collagenases, MMP-1, MMP-8, and MMP-13, are the dominant extracellular proteinases with the ability to cleave native fibrillar collagen types I, II, III, and V. As they can degrade dermal collagen, they are particularly relevant in melanoma [81]. MMP-1, secreted by melanoma and activated stromal cells, contributes to the progression of melanoma in two ways. Firstly, it promotes invasion and metastasis of melanoma by breaking down interstitial collagens. Secondly, it promotes tumor invasion and vascularization via protease activator receptor-1 (PAR-1)-induced gene expression [81,94]. In addition to melanoma, PAR-1, an oncogene, is highly expressed in many types of cancer. The expression of PAR-1 correlates with the depth of melanoma invasion [81]. Hence, the process of tumor cell invasion requires a combination of PAR-1 activity and MMP-1 expression, both of which are responsible for the collagenolytic function.

MMP-13 is reported to be involved in the invasive VGP of melanomas. Its expression in stromal cells immediately adjacent to the tumor is higher upon tumor cell invasion [82,92]. An increased risk of malignant melanoma is correlated with the expression of MMP-8 [92].

MMP-3 can activate pro-MMPs, such as pro-MMP-1 and pro-MMP-13. It is expressed in metastatic melanoma and is correlated with shorter disease-free survival [92,95]. MMP-7 or matrilysin is expressed and produced by primary cutaneous and metastatic melanomas [92,96]. Matrilysin is secreted as a pro-enzyme and is activated extracellularly. Its expression in melanomas enhances tumor cell growth and metastasis, thereby reducing the survival rate [96]. Levels of MMP-12 correlates with melanoma cell invasion, lymph node metastasis, and tumor metastasis. It is a potential predictive marker for the prognosis of patients with melanoma [97].

The knowledge of events that are associated with the initiation and progression of melanoma could be used critically for the development of novel therapeutic agents aimed at increasing the effectiveness of cancer therapy and improving the survival rate of cancer patients. 

## 5. Conclusions

Cutaneous exposure to UV irradiation causes the up-regulation of several different MMPs that virtually impair the various components of the ECM. These alterations in the ECM are known to cause skin wrinkling, a major characteristic of premature skin aging. Regulation of MMPs is one of the strategies to prevent photodamage to the skin as their activities contribute to wrinkle formation. Use of free radical scavengers or antioxidants is considered on a therapeutic basis to diminish the damaging effects of UV radiation and to prevent UV-initiated photoaging because of their ability to inhibit the expression and activity of MMPs. 

MMPs play an important role in tumor development, growth, angiogenesis, and metastasis. Each of these proteinases has specific roles in determining the invasive capacity of the tumor. Hence, the function of distinct MMPs and their regulation should be considered the principal targets for development of antineoplastic drugs or chemotherapeutic agents.

## Figures and Tables

**Table 1:** Classification of human metalloproteinases (MMPs) and their function in relation to photoaging and photocarcinogenesis.

MMP Subgroup	MMP Number	Alternate Name	Role in Photoaging	Role in Photocarcinogenesis
**Collagenases**	MMP-1	- Interstitial collagenase -Type I Collagenase	- Collagen type I and III degradation	- Tumor growth in BCC and SCC - Facilitate tumor invasion in melanoma
	MMP-8	- Neutrophil collagenase	- Limited role	- Limited role in BCC and SCC - Increased risk of malignant melanoma
	MMP-13	- Collagenase-3	- Limited role	- Tumor invasion and angiogenesis in BCC and SCC - Involved in invasive VGP of melanoma
**Gelatinases**	MMP-2	- Gelatinase-A - 72 kDa type IV collagenase	- Collagen type IV degradation	- Growth initiation and tumor invasion in BCC and SCC - Hematogenous metastasis in melanoma
	MMP-9	- Gelatinase-B - 92-kDa type IV collagenase	- Degrade collagen type IV	- Growth initiation and tumor invasion in BCC and SCC - Related to the RGP of melanoma and tumor angiogenesis
**Stromelysins**	MMP-3	- Stromelysin-1 - Proteoglycanase - Transin-1	- Collagen type I degradation - Activate MMP-1, -7, and -9	- Tumor progression and metastasis in SCC - Activate pro-MMPs in melanoma
	MMP-10	- Stromelysin-2 - Transin-2	- Activate pro-MMPs	- Tumor initiation in SCC
	MMP-11	- Stromelysin-3	-	-
**Matrilysins**	MMP-7	- Matrilysin-1 - Pump-1	- Elastin degradation	- Tumor invasion
	MMP-26	- Matrilysin-2 - Endometase	-	-Limited role in BCC -Activate MMP-9 and promote tumor growth in SCC
**Membrane-type**	MMP-14	- MT1-MMP	-	- Tumor invasion - Activate MMP-2 - Tumor angiogenesis in melanoma
	MMP-15	- MT2-MMP	-	- Tumor invasion in melanoma
	MMP-16	- MT3-MMP	-	- Tumor invasion in melanoma
	MMP-17	- MT4-MMP	-	-
	MMP-24	- MT5-MMP	-	-
	MMP-25	- MT6-MMP	-	-
**Other types**	MMP-12	- Metalloelastase	- Elastin degradaion	- Tumor invasion, lymph node metastasis in melanoma
	MMP-19	- RASI-1	-	-
	MMP-20	- Enamelysin	-	-
	MMP-21	-	-	-
	MMP-22	-	-	-
	MMP-23	-	-	-
	MMP-28	- Epilysin	-	-

**Table 2:** Role of UV-A and UV-B in photoaging induced by MMPs.

MMPs	UV-A	UV-B
Collagenases		
MMP-1	+	++
MMP-8	NA	NA
MMP-13	+	+
Gelatinases		
MMP-2	+	+
MMP-9	+	+
Stromelysins		
MMP-3	+	++
MMP-10	+	++
Matrilysins		
MMP-7	+	+
MMP-26	NA	NA
MT-MMPs		
MMP-14	NA	NA
MMP-15,16	NA	NA
Other types		
MMP-12	++	+

Abbreviations: ++, highly upregulated; + upregulated; NA, no reported.

**Table 3:** Protective agents against photoaging.

Mechanisms	Protective Agents
MMP inhibitors	*Galla chinensis*
	*Neonauclea reticulate*
	*Coffea Arabica*
	*Ixora parviflora*
Free radical scavengers	*Coffea Arabica*
	*Terminalia catappa*
	*Emblica officinalis*
	Epigallocatechin-3-gallate (EGCG)
	*Gynura procumbens*
	*Caesalpinia sappan* L.

## Abbreviations

^1^O_2_	Singlet oxygen
AP-1	Activator protein 1
BCC	Basal cell carcinoma
bFGF	Basic fibroblast growth factor
COX-2	Cyclooxygenase 2
CXCR4	C-X-C chemokine receptor type 4
ECM	Extracellular matrix
EGCG	Epigallocatechin gallate
EGFR	Epidermal growth factor receptor
EMT	Epithelial-mesenchymal transition
ERK	Extracellular signal-regulated kinase
GM-CSF	Granulocyte macrophage colony stimulatory factor
H_2_O_2_	Hydrogen peroxide
HB-EGF	Heparin-binding epidermal-like growth factor
HIF-1α	Hypoxia-inducible factor-1α
IL-1α	Interleukin 1 alpha
IL-6	Interleukin 6
JNK	c-Jun NH2-terminal kinase
kDa	Kilodalton
LN	Lymph node
MAPK	Mitogen-activated protein kinase
MMPs	Matrix metalloproteases
MT-MMP	Membrane-type matrix metalloprotease
NEP	Neutral endopeptidase
NF-κB	Nuclear factor-kappa B
O_2_−	Superoxide anion
OH groups	Hydroxyl group
OH.	Hydroxyl radical
PAR-1	Protease activator receptor 1
PDGF	Platelet-derived growth factor
RGP	Radial growth phase
ROS	Reactive oxygen species
SCC	Squamous cell carcinoma
SDF-1	Stromal cell-derived factor 1
TAMs	Tumor-associated macrophages
TCF	T-cell transcription factor
TGF-β	Transforming growth factor-beta
TIMP	Tissue inhibitors of matrix metalloprotease
TNF-α	Tumor necrosis factor alpha
UV	Ultraviolet
VEGF	Vascular endothelial growth factor
VGP	Vertical growth phase
